# 

**Published:** 1860-04

**Authors:** 


### CONTENTS

OF THE

### Chicago Medical Journal for April, 1860.

### ORIGINAL COMMUNICATIONS.

PAGE.

ARTICLE 1.—Strangulated Hernia, Treated by Opium. By S. W. Noble, M. D. .......... 185
ARTICLE 2.—Physiological Incest. By W. Boyd Powell, M. D., Covington, Ky. .......... 196
ARTICLE 3.—Stomatitis Materna. By L. S. Ellis, M. D., Chicago .......... 200
ARTICLE 4.—Blount on Opium .......... 209
ARTICLE 5.—Tape Worms. By E. C. De Puy, M. D., of Freeport, Ill. .......... 213
ARTICLE 6.—Cases of Re-section of the Upper Jaw. By Daniel S. Brainard, M. D. .......... 215
Report of Meeting of the Chicago Academy of Medical Sciences, March 2d, 1860 .......... 222

### SELECTIONS.

Variola and Vaccinia .......... 225
Do Bad Smells Cause Disease? .......... 233

### EDITORIAL.

Book Notices .......... 237 to 245
New Medical Journals .......... 245
Chilblains .......... 245
Typhoid Fever. By "A." .......... 246
American Medical Association .......... 246
Illinois State Medical Society, and the National Association .......... 247
Letter from "Medicus," .......... 248

